# *Chlamydiaceae* in wild, feral and domestic pigeons in Switzerland and insight into population dynamics by *Chlamydia psittaci* multilocus sequence typing

**DOI:** 10.1371/journal.pone.0226088

**Published:** 2019-12-30

**Authors:** Prisca Mattmann, Hanna Marti, Nicole Borel, Martina Jelocnik, Sarah Albini, Barbara Renate Vogler

**Affiliations:** 1 National Reference Centre for Poultry and Rabbit Diseases (NRGK), Institute for Food Safety and Hygiene, Vetsuisse Faculty, University of Zurich, Zurich, Switzerland; 2 Swiss Ornithological Institute, Sempach, Switzerland; 3 Institute of Veterinary Pathology, Vetsuisse Faculty, University of Zurich, Zurich, Switzerland; 4 Genecology Research Centre, University of the Sunshine Coast, Sippy Downs, Australia; Tokat Gaziosmanpasa University, TURKEY

## Abstract

Feral pigeons, common wood pigeons and Eurasian collared doves are the most common representatives of the *Columbidae* family in Switzerland and are mostly present in highly populated, urban areas. Pigeons may carry various members of the obligate intracellular *Chlamydiaceae* family, particularly *Chlamydia (C*.*) psittaci*, a known zoonotic agent, and *C*. *avium*. The objective of the study was to identify the infection rates of common free-roaming pigeons for different Chlamydia species with the overall aim to assess the risk pigeons pose to public health. In this study, 431 pigeons (323 feral pigeons, 34 domestic pigeons, 39 Eurasian collared doves, 35 common wood pigeons) from several geographic locations in Switzerland were investigated for the presence of *Chlamydiaceae*. Samples consisted of pooled choanal-cloacal swabs (n = 174), liver samples (n = 52), and paired swab and liver samples from 205 pigeons (n = 410). All 636 samples were screened using a *Chlamydiaceae* family-specific 23S rRNA real-time PCR (qPCR). Subsequent species identification was performed by DNA-microarray assay, sequencing of a 16S rRNA gene fragment and a *C*. *psittaci* specific qPCR. In total, 73 of the 431 pigeons tested positive for *Chlamydiaceae*, of which 68 were positive for *C*. *psittaci*, four were *C*. *avium*-positive and one pigeon was co-infected with *C*. *avium* and *C*. *psittaci*. The highest infection rates were detected in feral (64/323) and domestic pigeons (5/34). Common wood pigeons (2/35) and Eurasian collared doves (2/39) revealed lower infection rates. Additionally, multilocus sequence typing of twelve selected *C*. *psittaci*-positive samples revealed closely related sequence types (ST) between and within different Swiss cities. Furthermore, liver and corresponding swab samples from the same bird were colonized by the same ST. Considering the high infection rates of *C*. *psittaci* in domestic and feral pigeons, close or frequent contact to these birds poses a human health risk.

## Introduction

Members of the *Chlamydiaceae* family are gram negative, obligate intracellular bacteria with a biphasic developmental cycle. The single genus *Chlamydia* (*C*.) consists of thirteen species and three *Candidatus* species [1–3]. The most well-known chlamydial species harboured by birds is *C*. *psittaci*, which has been reported in at least 467 bird species belonging to 30 different orders [4]. This pathogen causes asymptomatic to severe systemic infections in several bird species, depending on susceptibility of the host species, immune status, infectious dose and virulence of the strain involved [5]. Transmission can occur by inhalation of contaminated dust, feather particles and respiratory tract secretions [6]. In birds, bacterial shedding can be intermittently activated by stressful events such as breeding, migration or other illnesses, without presentation of clinical symptoms [5].

*C*. *psittaci* is a zoonotic agent causing ornithosis, an influenza-like illness in humans, potentially leading to atypical pneumonia with sometimes fatal outcome [7]. Humans contract disease during close contact with infected birds by inhalation of respiratory secretions or dust from dried feces [6].

Based on the outer membrane protein A (*omp*A), *C*. *psittaci* is divided into nine genotypes and several subtypes, which are more or less associated with different hosts. Seven of these genotypes are generally found in avian hosts (A-F and E/B) [8–11]. Genotypes A and F are primarily found in psittacine birds, B in pigeons, C in ducks and geese, and D in turkeys. Genotype E infects a broad range of birds including pigeons [11], while E/B has been described in ducks [9]. Human infections are most frequently associated with genotype A, causing more severe infections than other genotypes [12–15].

*C*. *avium*, another chlamydial species infecting birds, was first described in 2014 [16] and has so far been reported in feral pigeons from Italy, France, Germany and the Netherlands, in a parrot from Germany and in a mallard from Poland [16–18]. To date, it is still unclear whether it causes disease in birds, if it has zoonotic potential or how it is transmitted.

Pigeons may become infected with several chlamydial species, including *C*. *psittaci* (the most common *Chlamydia* species identified in pigeons), *C*. *avium*, *C*. *abortus*, *C*. *pecorum* and *C*. *trachomatis* [19]. In Swiss feral pigeons, *C*. *psittaci* is the only species of the family *Chlamydiaceae* identified to date [20–22] and in general, research in avian *Chlamydia* seems to focus on *C*. *psittaci* and feral pigeons. Worldwide, several studies on *C*. *psittaci* in feral pigeons have been conducted, revealing a seroprevalence of up to 95.6%, while chlamydial DNA could be detected in up to 50% of the tested pigeons [23]. Especially in cities, where feral pigeons find easy access to food sources, they can build large populations of more than 300–400 pigeons per km^2^, leading to more stressed and diseased birds and thus to an increased risk for pathogen transmission to humans [5, 24]. Additionally, the close contact to feral pigeons through feeding, or even briefly passing areas with a high pigeon density, may increase the likelihood for zoonotic transmission of *C*. *psittaci* [23]. Whether any of the other *Chlamydiaceae* harboured by pigeons, apart from *C*. *psittaci*, may cause psittacosis-like disease in humans is not known.

The Swiss feral pigeon population has been stable or, in some areas, slightly declining in the last 20 years [25], due to different population management programs, e.g. culling schemes or reduction of food availability. In 2001, Lucerne introduced a population management program [26, 27] primarily focusing on banning public feeding and building two pigeon lofts to attract the birds. Droppings accumulating in the lofts are disposed resulting in a decreased fecal load in the city decreasing the risk of disease transmission to humans. In 2012, a different population management program was introduced in Berne consisting of catching all available pigeons, followed by euthanasia of clinically unhealthy birds and endoscopic sterilization of males. In addition, all caught pigeons were ringed and placed in one of five pigeon lofts. Both of these city loft programs were successful: the pigeon population decreased from around 7’000 individuals in 2001 to 2’500 in 2015 in Lucerne and from around 10’000 in 2011 to currently 1’500 birds in Berne [27, 28]. In Zurich, the feral pigeon population is managed primarily by culling. In 2019, an estimated 16’000 feral pigeons lived in the city of Zurich [29].

Switzerland is home to five different species of free-roaming pigeons. In detail, the four wild pigeon species common wood pigeon (*Columba palumbus*), stock dove (*Columba eonas*), Eurasian collared dove (*Streptopelia decaocto*), and European turtle dove (*Streptopelia turtur*), and feral pigeons (*Columba livia domestica*). In areas inhabited by humans, feral pigeons, common wood pigeons and Eurasian collared doves are well documented with a tendency towards increasing populations for the last two species [25]. These three pigeon species are potential hosts for *Chlamydia psittaci* [4] and possibly other *Chlamydia* species. However, there is no data available about the presence of *C*. *psittaci* and other *Chlamydia* species in Swiss wild pigeons. The present study aimed at collecting baseline data on the presence of *Chlamydiaceae* in three different free roaming Swiss pigeon species (feral and domestic pigeons, common wood pigeons and Eurasian collared dove), with insights into the population genetics of *C*. *psittaci* by using typing schemes such as *omp*A genotyping and multilocus sequence typing (MLST) [30, 31].

## Materials and methods

### Samples

A total of 636 samples from 431 pigeons belonging to the three species i) *Columbia livia domestica*, i.e. domestic (homing pigeons, fancy pigeons, flying/sporting pigeons) and feral pigeons (“city pigeons”), ii) *Streptopelia decaocto* (Eurasian collared dove) and iii) *Columba palumbus* (common wood pigeon) from different geographical areas in Switzerland between May 2014 and October 2018 were analyzed. Individual samples consisted of combined choanal/cloacal swabs (c/c-swabs; n = 174) and liver samples (n = 52). Additionally, paired samples of c/c-swab and liver (n = 107), and cloacal swab (c-swab) and liver (n = 98) were available (Table 1). Samples derived from the diagnostic service of the National Reference Centre for Poultry and Rabbit Diseases (NRGK) and originated from birds found at various locations admitted to the rehabilitation center of the Swiss Ornithological Institute based in the Canton of Lucerne (n = 58) and from feral pigeons inhabiting three of the five pigeon lofts in Berne (loft A, n = 25; loft B, n = 49; loft C, n = 23). All loft birds were tested on the same day excluding repeated sampling of individuals. Additional samples from feral pigeons culled by the game warden in the context of the local population control program in the city of Zurich and surrounding areas (greater Zurich area) (n = 142) completed the sample set (Table 1). The majority of the rehabilitation center pigeons were found in rural regions, like small villages or farmland (Table 1). Upon collection and until DNA extraction, the swabs and liver samples were stored at -20°C, samples from Zurich were stored at -80°C. A complete list of samples is provided in S1 Table.

**Table 1:** Number of pigeons according to type of pigeon, sample material and place of origin.

	Single samples		Paired samples		Place of origin				Total no. of pigeons
	C/c-swab	Liver	C/c-swab* + liver	C-swab** + liver	Berne	Greater Lucerne area**	Greater Zurich area	Various***	
Feral pigeon	142	47	36	98	123	23	142	35	323
Domestic pigeon	17	2	15	0	0	8	0	26	34
Eurasian collared dove	12	1	26	0	0	2	3	34	39
Common wood pigeon	3	2	30	0	0	13	1	21	35
Total	174	52	107	98	123	46	146	116	431

*C/c-swab = combined choanal/cloacal swab; **C-swab = cloacal swab

**Lucerne, Kriens, Horw, Emmen, Emmenbruecke, Rothenburg

***More rural places as compared to the city areas within the cantons Lucerne, Obwalden, Nidwalden, Schwyz, Zug, Aargau, Solothurn, Zurich, Schaffhausen, Basel-Land, Thurgau and St. Gallen

### DNA extraction

DNA was extracted using a commercial kit (Genomic DNA from tissue, *NucleoSpin*^*®*^ *Tissue* from *Macherey-Nagel*, Düren, Germany) according to the manufacturer’s instructions. For each extraction lot, a negative extraction control was prepared by using “Buffer T1” instead of the sample. Extracts were stored at -20°C until further analysis was performed. Extracted DNA was measured on a Nanodrop 2000c spectrophotometer (Thermo Fisher Scientific, Waltham, MA, USA) to determine DNA quantity and quality (260/280 value).

### *Chlamydiaceae* screening of extracted DNA

**Fig 1:**
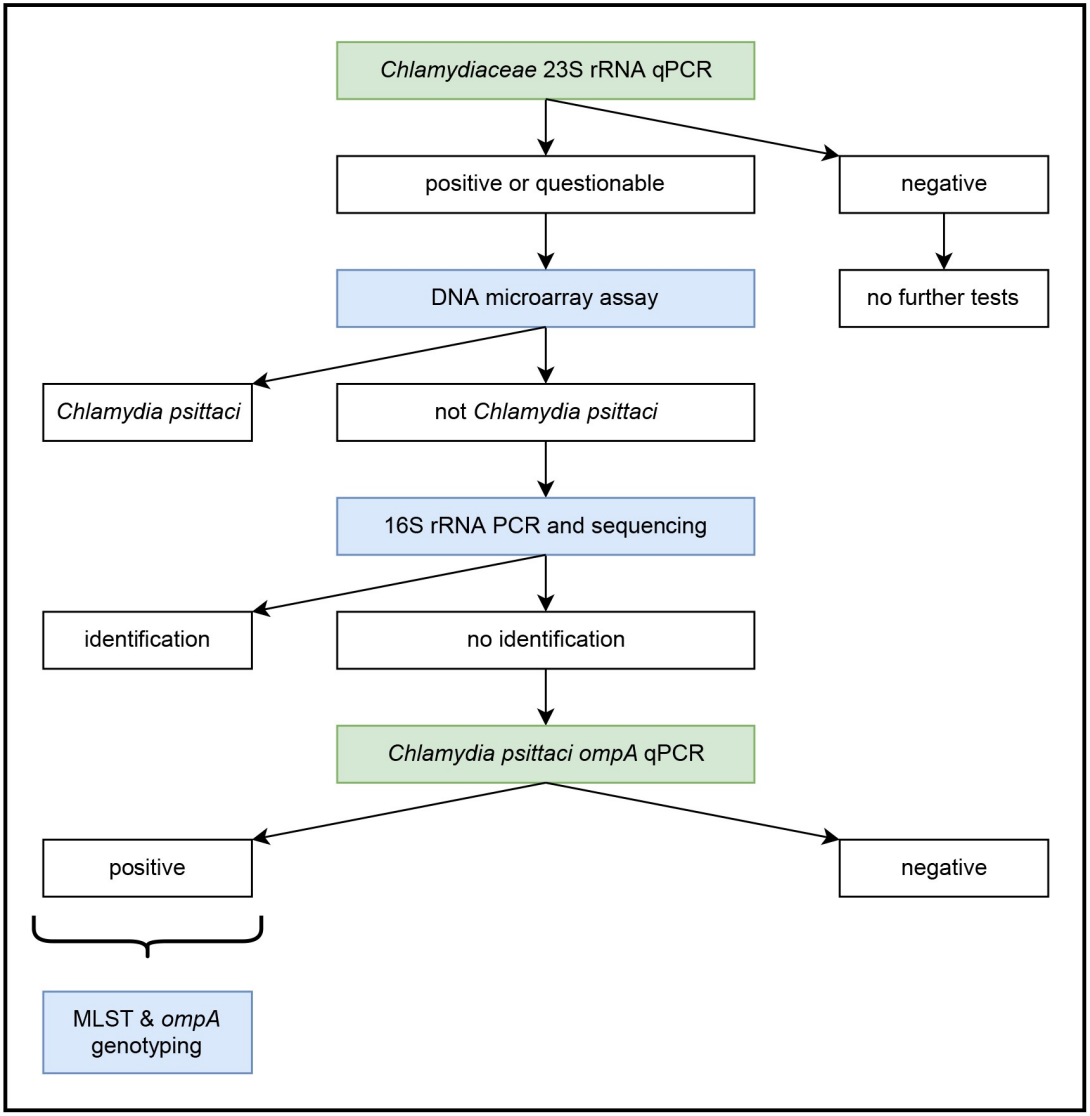
Decision tree for step-wise typing of samples originating from pigeons. Green-colored boxes mark methods using quantitative PCRs, while conventional PCRs are colored in blue. Out of 86 *C*. *psittaci*-positive samples, 12 selected samples were further characterized by multilocus sequence typing (MLST) and *ompA* genotyping.

All extracted DNA samples (n = 636) were investigated according to the decision tree as depicted in Fig 1. All primers and probes used in this study are listed in Table 2.

**Table 2:** Primers and probes and their final concentration (nM) in the PCR reagent mix for different quantitative PCR (qPCR) and conventional PCR (PCR) tests used in this study.

Method	Target	Primer & Probe	Sequence (5’-3’)	Final conc. of primers and Probes in the PCR reagent mix	Amplicon size (base pairs)	Annealing temperature (°C)	references
*Chlamydiaceae* 23S rRNA qPCR	23S rRNA	Ch23S-F	CTGAAACCAGTAGCTTATAAGCGGT	500 nM	111	60	Ehricht et al. [32]
		Ch23S-R	ACCTCGCCGTTTAACTTAACTCC				
		Ch23S-p	FAM-CTCATCATGCAAAAGGCACGCCG-TAMRA	200 nM			
	eGFP	eGFP-1-F	GACCACTACCAGCAGAACAC	200 nM	177		Hoffmann et al. [33]
		eGFP-10-R	CTTGTACAGCTCGTCCATGC				
		eGFP-HEX	HEX-AGCACCCAGTCCGCCCTGAGCA-BHQ1				
DNA microarray assay PCR	23S rRNA	U23F-19	ATTGAMAGGCGAWGAAGGA	500 nM	171	50	Ehricht et al., Borel et al. [32, 34]
		23R-22	biotin-GCYTACTAAGATGTTTCAGTTC				
	eGFP	eGFP-11-F	CAGCCACAACGTCTATATCATG	50 nM	276		Hoffmann et al. [33]
		eGFP-10-R-Bio	Bio-CTTGTACAGCTCGTCCATGC				
16S rRNA PCR	16S rRNA	16S IGF (short)	GATGAGGCATGCAAGTCGAACG	300 nM	278	58	Pospischil et al. [35]
		16S IGR (short)	CCAGTGTTGGCGGTCAATCTCTC				
*C*. *psittaci omp*A qPCR	*omp*A	CppsOMP1-F	CACTATGTGGGAAGGTGCTTCA	900 nM	76	60	Pantchev et al. [36]
		CppsOMP1-R	CTGCGCGGATGCTAATGG				
		CppsOMP1-S	FAM-CGCTACTTGGTGTGAC-TAMRA	200 nM			
	eGFP	eGFP-1-F	GACCACTACCAGCAGAACAC	900 nM	132		Hoffmann et al. [33] PCR modified by Blumer et al [37]
		eGFP-2-R	GAACTCCAGCAGGACCATG				
		eGFP-Hex	HEX-AGCACCCAGTCCGCCCTGAGCA-BHQ1	200 nM			
MLST PCR	*eno*A	YPenoA3	CCTATGATGAATCTCATTAATGG	200 nM	450–500	53	Pannekoek et al. [31]
		YPenoA4	CCCAACCATCAAAATCTTCTTCCG				
	*fum*C	YPfumC1	GGGCTCCTGAGGTTATGCC		500–600	53	
		YPfumC2	CGCAAATAATGAATCACCTTATC				
	*gat*A	YPgatA3	GCCTTAGAGTTAAGAAATGCCG		500–600	60	
		YPgatA4	CCCCCTGTATCGGAACCTAACGC				
	*gid*A	YPgidA1	GCTTATTAGAGAGCTGTCCTGGC		500–670	53	
		YPgidA2	CGCGTTTTCTAACCCACGG				
	*hem*N	YPhemN1	GGATCCATTTCGGAGGAGGC		500–630	53	
		YPhemN2	CCTGAAAGGATTTTCTCATGG				
	*hflX*	YPhflX3	GAGATTTTTGCTAATCGAGCG		500–610	53	
		YPhflX4	GTAAAACATCTTCATGTAACGC				
	*opp*A	YPoppA3	ATGCGCAAGATATCAATGGG		500–610	60	
		YPoppA4	GGCAAGGTTTGGTGTAACTCGC				
*omp*A PCR	*omp*A	ompA F (CTU)	ATGAAAAAACTCTTGAAATCGG	200 nM	1200	49	Sachse et al. [30]
		ompA rev	TCCTTAGAATCTGAATTGAGC				

### Quantitative and conventional PCRs

All quantitative PCRs (qPCR; Fig 1, Table 2) were run on an Applied Biosystems^®^ 7500 Real-Time PCR System (Thermo Fisher Scientific). As internal amplification control, eGFP was added to the reaction mix [33].

Products from all conventional PCRs (Fig 1, Table 2) were purified using the QIAquick^®^ PCR Purification Kit (Qiagen) according to the manufacturer’s instructions.

Purified amplicons were Sanger sequenced by Microsynth (Balgach, Switzerland) [38]. The obtained sequences were assembled and analyzed using the CLC Main Workbench 8 software and compared against the NCBI database using the BLASTn tool (NCBI, https://blast.ncbi.nlm.nih.gov/) or the MLST database (http://pubmlst.org/chlamydiales/).

### *Chlamydiaceae* qPCR

The samples were analyzed with the 23S rRNA-based *Chlamydiaceae* family-specific qPCR [32] as modified by Blumer et al. [37]. The cycle conditions were 95°C for 20 s, followed by 45 cycles of 95°C for 3 s, and 60°C for 30 s. All samples were tested in duplicate and the cycle threshold was set at 0.1 in each run. A sevenfold dilution series of *C*. *abortus* DNA with a known number of DNA copies was included in each run as a standard curve allowing the software of the Applied Biosystems^™^ 7500 Real-Time PCR System to calculate the number of copies in positive samples of that run. Molecular grade water was used as a negative control. Samples were interpreted as positive if the mean cycle threshold (Ct value) was < 38. Samples with higher Ct values or inhibited amplification were re-tested in duplicate. Samples repeatedly showing a Ct value > 38 were considered as positive. The *Chlamydiaceae* copy number per μl was determined directly by the PCR instrument using the standard curve, calculating the percentage of *Chlamydia*-DNA out of total DNA (*Chlamydia*-%).

### DNA microarray assay

The sample DNA, including internal control DNA (Intype IC-DNA, Qiagen Labor, Leipzig, Germany), was amplified and biotin labelled using the method described by Borel et al. [34]. The cycle conditions were 96°C for 10 min, followed by 39 cycles of 94°C for 30 s, 50°C for 30 s and 72°C for 30 s and a last step of 72°C for 4 min. The labelled DNA was hybridized using the Hybridization Kit 245200100 (Alere Technologies GmbH; now Abbott, Chicago, Illinois, USA) and analyzed using the ArrayStrip^™^ system (ChlamType-23S AS-4 Kit, Alere Technologies GmbH, Jena, Germany), as established by Borel et al [34]. With the current kit, eleven *Chlamydia* species and nine *Chlamydia*-like organisms can be identified.

### 16S ribosomal RNA (rRNA) PCR

The conventional 16S rRNA PCR was performed as described by Pospischil et al. [35], using the primers 16S IGF (short) and 16S IGR (short) to amplify a sequence of 298 base pairs (bp) (Table 2).

Per sample, a 50 μl reaction mix was prepared, containing 1 μl (< 150 ng/μl) sample template, 1x PCR buffer with MgCl_2_ (Roche Diagnostics GmbH), 0.5 mM MgCl_2_ Stock Solution (Roche Diagnostics GmbH), 0.2 nM dNTP (PCR Nucleotide Mix, Roche Diagnostics GmbH), 300 nM of both primers and 0.02 U/μl FastStart Taq DNA Polymerase.

Cycling conditions were 95°C for 5 min, followed by 40 cycles of 95°C for 60 s, 65°C for 60 s and 72°C for 90 s and a final extension of 72°C for 10 min.

### *C*. *psittaci* quantitative PCR

The *C*. *psittaci*-specific qPCR was performed according to the protocol as described by Pantchev et al. [36]. The reaction mix contained 4 μl (< 150 ng/μl) sample template, 1 μl eGFP template, 1x TaqMan Universal PCR MasterMix, 900 nM of the primers CppsOMP1-F and CppsOMP1-R, 200 nM probe CppsOMP1-S, 900 nM of the primers eGFP-1-F and eGFP-2-R and 200 nM probe eGFP-HEX [33] in a final volume of 25 μl.

### *C*. *psittaci* typing

Twelve *C*. *psittaci* strains were selected for further characterization by performing *C*. *psittaci*-specific multilocus sequence typing (MLST) as described by Pannekoek et al. [31] and genotyping based on the major outer membrane protein (*omp*A) gene [30]. Selection aimed at creating a sample subset that represents the diversity of the main sample set. It was based on (I) geographical location (city, pigeon loft), (II) pigeon species and (III) positivity in a set of paired samples. Samples finally included were: swab samples of five feral pigeons from the lofts A, B, and C in Berne (1, 2, and 2 pigeons, respectively), two feral pigeons from Zurich, and two feral pigeon from the greater Lucerne area; a matching liver sample to one of the feral pigeons from the greater Lucerne area; a swab sample of a domestic pigeon from the greater Lucerne area, and one swab sample from an Eurasian collared dove from Inwil. To make sure that MLST analysis will be successful, only samples wih a *Chlamydia*-% above 0.002 were selected. Thus, a sample from a common wood pigeon could not be included.

### Multilocus sequence typing

**Fig 2:**
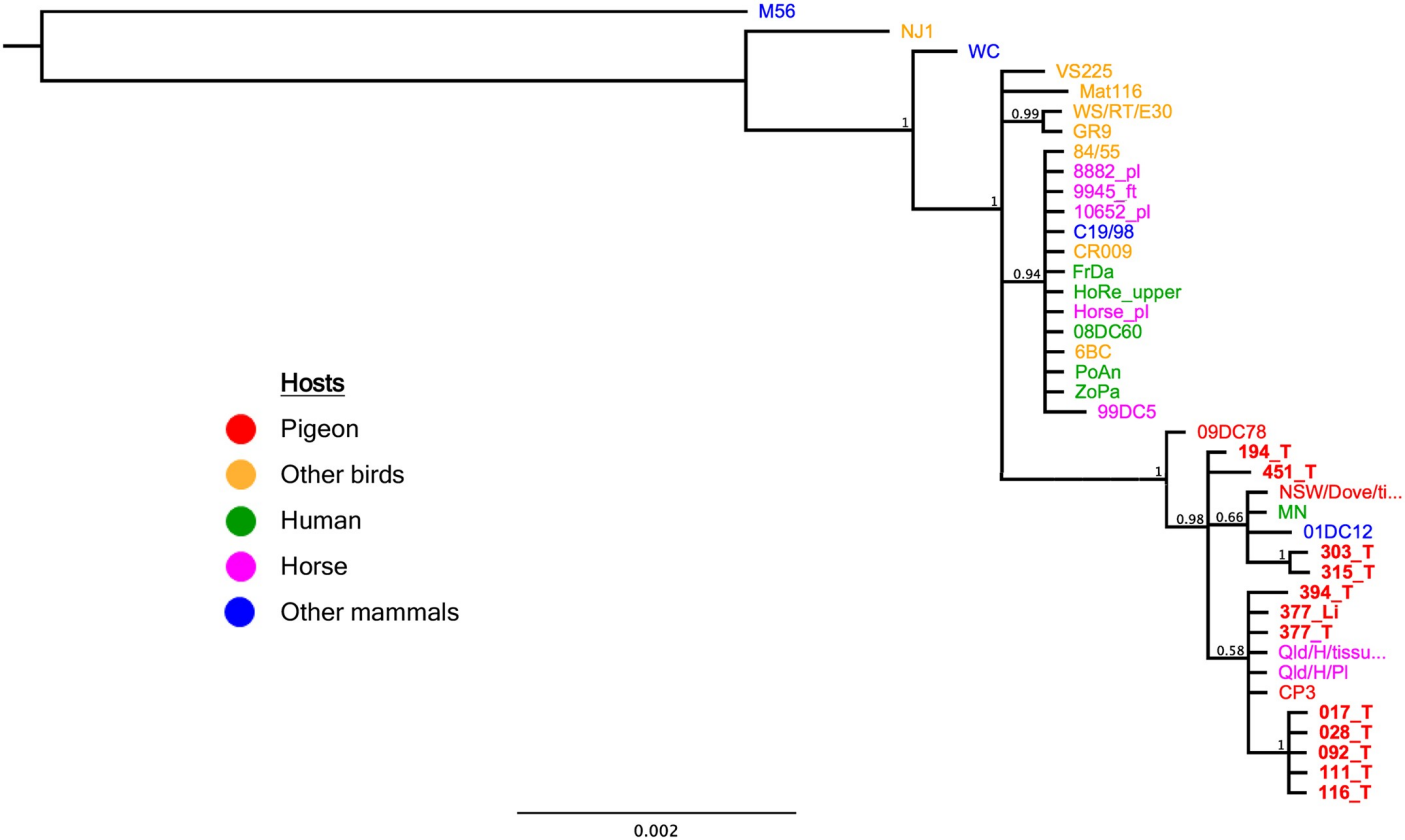
Bayesian phylogenetic tree of concatenated multilocus sequence typing (MLST) sequences from 40 *Chlamydia psittaci* strains from avian and mammalian hosts. The host is color-labeled as depicted in the legend. M56 taxa was used as an outgroup. Samples from this study are marked in bold letters. The posterior probability values are displayed on the tree nodes.

The conventional PCRs targeting seven housekeeping genes were performed as previously described [31]. For each sample, a 50 μl reaction mix was used, containing 1x AmpliTaq Gold^™^ 360 Master Mix (Thermo Fisher Scientific), 200 nM of each primer (Table 2) and 3 μl sample template with a DNA concentration of 25 ng/μl. Cycling conditions were 95°C for 10 min, followed by 35 cycles of 95°C for 30 s, 53°C (*eno*A, *fum*C, *gid*A, *hem*N, *hfl*X) or 60°C (*gat*A, *opp*A) for 30 s, 72°C for 60 s and a final step at 72°C for 7 min. If amplification resulted in weak bands, a modified cycling protocol with 40 cycles of 95°C for 60 s, 53°C/60°C° for 60 s, 72°C for 90 s was used. Subsequently, the PCR products were analyzed as mentioned above. The alignments of the concatenated sequences (3098 bp) were generated using MAFFT as implemented in Geneious 11.0.5 [39]. The mid-point rooted Bayesian tree was constructed using the concatenated MLST 3098 bp MAFFT alignment with the MrBayes program (as implemented in Geneious). The tree parameters included: GTR +G nucleotide model, with 4 MCMC chains with million generations, sampled every 1000 generations and with the first 100 000 trees discarded as burn-in. The additional strains used for Bayesian analysis as shown in Fig 2 are listed in S2 Table. The MLST sequences generated in this study are deposited in PubMLST/Chlamydiales (https://pubmlst.org/chlamydiales/).

### *Omp*A genotyping

**Fig 3:**
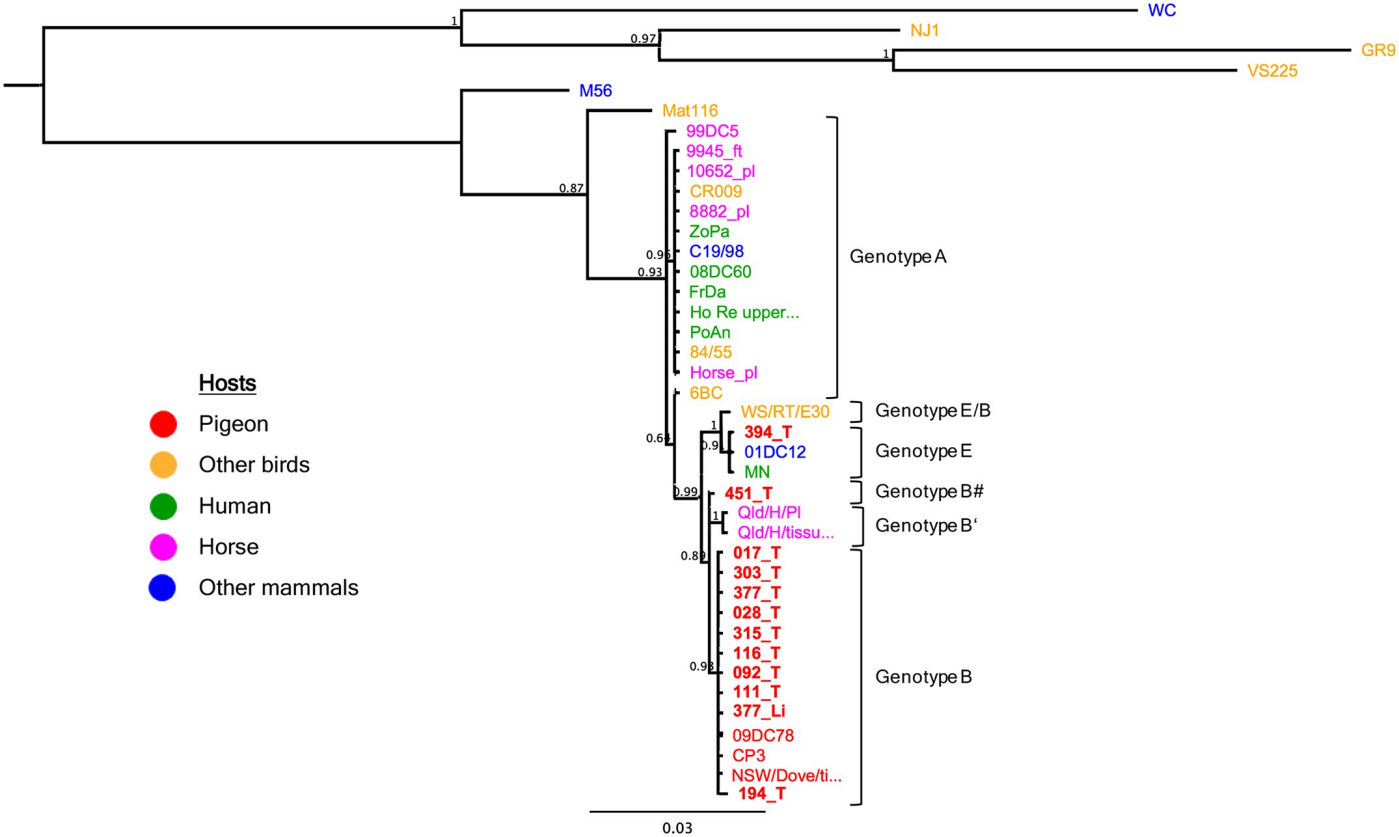
Bayesian phylogenetic tree of *omp*A gene sequences from 40 *Chlamydia psittaci* strains from avian and mammalian hosts. The host is color-labeled as depicted in the legend. M56 taxa was used as an outgroup. Samples from this study are marked in bold letters. The posterior probability values are displayed on the tree nodes.

Each reaction mix with a final volume of 50 μl contained 1x AmpliTaq Gold 360 master mix (Thermo Fisher Scientific), 200 nM of the primers CTU and ompA rev [30] and 3 μl sample template with a DNA concentration of 25 ng/μl. Cycling conditions were 10 min at 95°C, followed by 35 cycles of 95°C for 30 s, 49° for 30 s, 72°C for 60 s and a final elongation at 72°C for 7 min. If amplification resulted in weak bands, a modified cycling protocol with 40 cycles of 95°C for 60 s, 49° for 60 s, 72°C for 90 s was used. Alignments of the 1050 bp sequences and Bayesian analysis was performed as described above. The *C*. *psittaci* strains used for Bayesian analysis as shown in Fig 3 are listed in S2 Table. The sequences generated in this study are available in Genbank under accession numbers MK805041—MK805052.

### Ethics statement

Samples for this study included swab and organ samples of pigeons taken during necropsy and choanal/cloacal swab samples of live animals collected by a certified veterinarian for diagnostic use. Therefore, no approval of the ethics committee was sought.

## Results

### *Chlamydiaceae* PCR revealed an average infection rate of 16.9% in Swiss pigeons

A total of 70 swab (18.5%) and 22 liver samples (8.6%) of a total of 73 pigeons (16.9%) were positive for *Chlamydiaceae* (Table 3).

**Table 3:** Number of samples (positive/total) by pigeon species tested for *Chlamydiaceae*.

Species	Swabs only	Paired samples:		Liver only	Total no. of pigeons
		swabs	liver		
Feral pigeons	20/142	42/129	18/129	1/47	64/323
Domestic pigeons	4/17	1/15	0/15	0/2	5/34
Common wood pigeon	0/3	1/30	1/30	0/2	2/35
Eurasian collared dove	0/12	2/26	2/26	0/1	2/39
Total	24/174 (13.8%)	46/205 (22.4%)	21/205 (10.2%)	1/52 (1.9%)	73/431 (16.9%)

In feral pigeons, *Chlamydiaceae* infection rates differed according to geographical origin. While pigeons from the greater Zurich area showed an infection rate of 27.5% (39/142), pigeons from the greater Lucerne area and Berne showed lower overall infection rates of 15.5% (19/123) and 17.4% (4/23), respectively. The results from the pigeons of the three lofts within Berne, however, differed greatly with 4/23 (17.4%) *Chlamydiaceae*-positive pigeons in Loft C, but only 1/25 (4.0%) and 2/49 (4.1%) in Loft A and B. The latter infection rates were similar to the overall infection rate determined for feral pigeons from the more rural places of different cantons (Lucerne, Obwalden, Nidwalden, Schwyz, Zug, Aargau, Solothurn, Zurich, Schaffhausen, Basel-Land, Thurgau, St. Gallen), where only 2/35 (5.7%) individuals were positive for *Chlamydiaceae*. Similar infection rates again were detected in the Swiss wild pigeon species Eurasian collared dove (2/39; 5.1%) and common wood pigeon (2/35; 5.7%).

Different results were also detected for the different sample materials. A comparison of the results for the paired swab and liver samples (410 samples of 205 pigeons) revealed a total of 46/205 (22.4%) swab samples and 21/205 (10.2%) liver samples to be positive for *Chlamydiaceae*. These samples originated from 19 pigeons positive in both samples (swab and liver), 27 pigeons positive in the swab sample only and two pigeons positive in the liver sample only. In average, the chlamydial load (*Chlamydia*-%) in swab samples was 10^4^ times higher than in the matching liver sample with only one pigeon showing a higher *Chlamydia*-% in the liver sample compared to the associated swab sample (S3 Table).

### *C*. *psittaci* is the predominant chlamydial species in Swiss pigeons

All 92 *Chlamydiaceae* positive samples originating from 73 pigeons were further investigated by the Arraymate microarray assay. Of these, 53 samples (57.6%) of 37 individuals could be identified as *C*. *psittaci*, five samples (5.4%) of 4 individuals as *C*. *avium* and one (1.1%) as a mixed infection of *C*. *psittaci* and *C*. *avium*. The remaining 33 samples (35.9%) could not be further identified by microarray assay and were subjected to the conventional 16S rRNA PCR, as were the five *C*. *avium*-positive samples and the mixed-infected sample for confirmation reasons. Sequencing of PCR products was successful for 13 samples. Of those, the five samples from two domestic and two feral pigeons previously identified with a single infection with *C*. *avium* by microarray assay, were confirmed to be positive for *C*. *avium*, whereas the other eight samples from feral pigeons, including the one previously identified with a mixed infection of *C*. *avium* and *C*. *psittaci*, were identified as *C*. *psittaci*.

The 25 yet unidentified samples originating from 22 feral pigeons, two domestic pigeons, one common wood pigeon and one Eurasian collared dove, were further investigated with the *C*. *psittaci omp*A qPCR. All 25 samples were positive for *C*. *psittaci* (Table 4), including the samples with Ct values > 38 in the *Chlamydiaceae* qPCR.

**Table 4:** Number of samples (positive/total) for *Chlamydia* species detected in different pigeon species.

Species	*C*. *psittaci*	*C*. *avium*	*C*. *psittaci + C*. *avium*
Feral pigeons	61/323	2/323	1/323
Domestic pigeons	3/34	2/34	0/34
Common wood pigeon	2/35	0/35	0/35
Eurasian collared dove	2/39	0/39	0/39
Total	68/431 (15.8%)	4/431 (0.9%)	1/431 (0.2%)

### Different STs identified in different pigeon populations belong to *Omp*A genotype B and E

MLST analysis of twelve selected samples revealed that pigeons within one city were infected with *C*. *psittaci* strains from the same or closely related sequence types (ST). Between cities, however, the identified STs were distinct (Fig 2, S4 Table). Additionally, three new STs were detected due to a single nucleotide polymorphism (SNP) in the house keeping gene *hfl*X (ST212, feral pigeons from Zurich), a SNP in the house keeping gene *gat*A (ST216, Eurasian collared dove from Inwil) and due to a novel combination of house keeping gene alleles (ST213, Feral pigeon from Lucerne). Additionally, the analysis of the paired swab (377_T) and liver sample (377_Li) of one pigeon showed the same ST (ST27) in both samples (S4 Table).

*Omp*A typing of the twelve strains revealed, that 9/12 strains shared 100% sequence identity with the reference *C*. *psittaci* strain CP3 and thus belong to *omp*A genotype B. The strains identified as ST26 (194_T) and ST216 (451_T) both shared 99.9% sequence identity with the *C*. *psittaci* CP3 *omp*A sequence, showing a synonymous (A → G) single nucleotide polymorphism (SNP) on position 748 and a non-synonymous (A → G) SNP on position 466, and were denoted genotype B”and B#, respectively. Additionally, both strains showed 99.7% and 99.9% sequence identity with the novel genotype B’ detected in a horse from Queensland, Australia (strain Qld/H/Pl, Accession no. MG587894.1) (S4 Table). The strain identified as ST213 (394_T) belongs to genotype E, sharing 100% sequence identity with the previously described reference strain MN. Phylogenetic analysis of the *omp*A gene is shown in Fig 3.

## Discussion

### Different infection rates of feral pigeons in different geographical areas

The percentage of *Chlamydiaceae*-positive feral pigeons originating from Berne was notably higher in loft C (32.7%) as compared to lofts A and B (4.0% and 4.1%) although pigeons of all three lofts were kept under equal conditions. It can also be expected that the *C*. *psittaci* strains infecting the pigeons of the different lofts express similar virulence, since samples taken from five feral pigeons originating from these three different lofts were all typed as ST55 by MLST and *omp*A genotype B, the most common genotype found in pigeons [23, 40–42]. A possible explaination for the differences in infection rate might be the geographical location of the lofts with loft C being situated in the outskirts of Berne in close proximity to a forest and lofts A and B being located in an urban environment. However, distances to forest areas in Switzerland are relatively short: Switzerland is a small country with a total area of 41,285 km^2^ and one third covered by forests. Apart from bodies of water (>4%), and mountains above tree line, there is no area without any smaller or larger wood nearby.

While a study on feral pigeons from Lucerne from 2007 detected *Chlamydiaceae* DNA in 2/60 (3.3%) individuals, the present study, tested 4/23 (17.4%) of feral pigeons from the greater Lucerne area positive for *Chlamydiaceae*. Since the pigeons from the current study had been admitted to a wildlife hospital, sampled animals predominantly suffered from trauma or disease, which could have induced or increased chlamydial shedding due to stress [5].

In contrast to a previous study on feral pigeons from Zurich from 2008 [21], where 10/24 (42.7%) pigeons tested positive for *Chlamydiaceae*, the infection rate of 39/142 (27.4%) detected in the present study was much lower. It is unclear whether the amount of infected birds decreased over time or whether the difference is biased due to different sample sizes.

Since the infection rate seemed to be higher in the city of Zurich as compared to other Swiss cities (see above), which implemented different population management programs, the type of program might influence urban *Chlamydiaceae* epidemiology. The two population management programs in Berne and Lucerne were more effective than culling to achieve a smaller and healthier pigeon population. It was shown that culling, as performed in Zurich, only results in a temporary population size decrease; most likely due to the high compensatory potential of pigeons [43]. Additionally, the continuous extraction of animals by culling might result in an increased contact rate between individual pigeons due to the frequent restructuring of the population [44], thus leading to a potential increase of disease transmission events within the population.

The infection rate of *Chlamydiaceae*-positive feral pigeons (2/35, 5.7%) originating from rather rural geographical locations is comparable with the infection rate detected in wild pigeon species in the present study. This might be explained by the lower population density of feral pigeons in small villages and on farmland as compared to urban areas and which is comparable to that of common wood pigeons throughout Switzerland [25].

The infection rate of *C*. *psittaci* in feral pigeons varies widely between different European countries or cities, e.g. from 2.4% in Utrecht, the Netherlands [18], to 50% in Vinica, Republic of North Macedonia [23], and may even vary from year to year [19]. The infection rates seen in the greater Lucerne area and Berne are comparable to those of other cities in Europe [19, 23, 45]. Zurich on the other hand, shows one of the highest infection rates throughout Europe [23].

### Infection rates of domestic pigeons, Eurasian collared doves and common wood pigeons

Literature on *Chlamydiaceae* infection rates for free-roaming pigeons other than feral pigeons is scarce. In the present study, 5/34 (14.7%) domestic pigeons were positive for *Chlamydiaceae* which is comparable to infection rates reported in domestic pigeons elsewhere in Europe such as Slovenia with a infection rate of 16% [46] and Germany with 12.8–42.6% [47].

The infection rate of 2/39 (5.1%) Eurasian collared doves being positive for *C*. *psittaci* seems relatively low when compared to the results of a recent study on urban collared dove populations from Italy, where 46/76 (61%) were positive for *C*. *psittaci* [48]. However, comparison of results is difficult since birds from the present study originated from different locations, most-likely belonging to different colonies, while the Italian study analysed individuals from one population.

The analysis of common wood pigeons identified 2/35 (5.7%) individuals to be positive for *C*. *psittaci*; a similar infection rate (1/25, 4.0%) as detected by Sharples et al. [49] by *omp*B PCR in common wood pigeons in the UK.

### Molecular identification of *C*. *psittaci* strains and infection rate with *C*. *avium*

As expected, the present study revealed that *C*. *psittaci* is the predominant *Chlamydia* species identified in the three species of free-roaming Swiss pigeons with 68/73 *Chlamydiaceae*-positive pigeons positive for *C*. *psittaci*, 4/73 positive for *C*. *avium*, and 1/73 positive for both *Chlamydia* species. Confirmation by 16S rRNA PCR of *C*. *psittaci*, but not *C*. *avium* in the sample initially identified as mixed-infected might be explained by a higher copy number of *C*. *psittaci* in the sample, which was then predominantly amplified by the 16S rRNA PCR.

*C*. *psittaci* is the most common *Chlamydia* species identified in pigeons [19] and for Swiss feral pigeons the only *Chlamydia* species described so far [20–22].

*C*. *avium* was first described in 2014 [16]. So far, *C*. *avium* has been found in pigeons from France, Germany, Italy, and the Netherlands [16, 18]. In Germany, co-infections of *C*. *avium* and *C*. *psittaci* were detected in two young pigeons, both showing respiratory symptoms [16]. Szymańska-Czerwińska and Niemczuk [50] suggested that co-infection with *C*. *psittaci* and *C*. *avium* may cause clinical symptoms in birds. However, the pigeon tested positive for both of these *Chlamydia* species in the present study was euthanized due to an open metacarpal fracture. Apart from that, no clinical symptoms were observed. Although *C*. *avium* has been found mainly in pigeons, more recent studies reported *C*. *avium* infections in a parrot in Germany and a mallard duck in Poland [16, 17]. The zoonotic potential of *C*. *avium* is still unknown.

MLST analysis of selected *C*. *psittaci* strains revealed the same or closely related STs within a population, but distinct STs in different geographic locations, supporting the hypothesis that the exchange between different pigeon populations is limited [43, 44]. Therefore, it can be assumed that direct pathogen transmission between different populations is occurring only infrequently. Additionally, the same ST (ST27), *omp*A genotype B was detected in swab and liver of the same pigeon. A finding that was expected, indicating that infections are caused by a single genotype also being able to induce a systemic infection.

As expected, most *C*. *psittaci*-positive samples (9/12) that were further investigated by *omp*A sequencing were identical to the strain CP3 and thus belonged to genotype B, the genotype responsible for the majority of the *C*. *psittaci* infections in pigeons and thought to be endemic in the European pigeon population [23]. Two pigeons were infected by a strain showing 99.9% identity to strain CP3, but had a synonymous (sample 194_T) and a non-synonomous (sample 451_T) SNP compared to strain CP3 and were denoted *omp*A genotype B”and B#. Both strains additionally showed high identity (99.9% and 99.7%) with the strain Qld/H/Pl (Accession no. MG587894.1) isolated from the placenta of a horse abortion in Queensland, Australia. It may therefore be speculated that certain *C*. *psittaci* strains carried by birds have the potential to infect horses leading to abortion. One feral pigeon (394_T) was infected by a *C*. *psittaci* strain belonging to genotype E, which is frequently found in pigeons worldwide and has been detected in several other avian species from Europe and Asia, [23, 48, 51–54]. This genotype has been associated with several outbreaks in ducks and turkeys as well as fatal cases of chlamydiosis in ratites [6].

### Public health concerns

The detection of *C*. *psittaci*, a zoonotic agent, in all investigated pigeon species proves that not only feral and domestic pigeons may represent a potential public health hazard, but also wild pigeon species such as the common wood pigeon or Eurasian collared dove. Due to intermittent shedding, even clinically healthy pigeons or pigeons with a negative result in a PCR analysis might still be a source of infection [55, 56]. *Omp*A analysis of selected *C*. *psittaci* strains detected genotype B and E, both considered to be less pathogenic to humans than the closely related genotype A, predominantly found in psittacine birds [12, 14, 57, 58]. However, all genotypes of *C*. *psittaci* are considered zoonotic. A major risk factor identified for human *C*. *psittaci* infection was unprotected daily contact to domestic pigeons (i.e. contact to feather dust and fecal matter) [59].

## Conclusion

Of the four pigeon types investigated, feral pigeons showed the highest rate of infection with *Chlamydiaceae* (19.8%), followed by domestic pigeons (14.7%). While the identification of *Chlamydiaceae* in wild pigeon species from Switzerland was described for the first time, the infection rate was much lower (5.1% for Eurasian collared doves, 5.7% for common wood pigeons). *Chlamydia psittaci* was the most common *Chlamydia* species detected and the presence of *C*. *avium* in Switzerland was reported for the first time.

## Supporting information

S1 Table Comprehensive list of samples collected, analyses performed and according results. (n.a.—not available, n.d.—not done, n.i.—not identified).
(XLSX)

S2 Table *Chlamydia psittaci* strains shown in Figs 2 and 3. These strains are either *Chlamydia psittaci* reference strains or were isolated from horse abortions, humans showing severe respiratory symptoms and from birds originating from Europe and Australia. Additionally, strains isolated from other mammals from Germany are listed.
(XLSX)

S3 Table Percentage of *Chlamydia* DNA out of total DNA (*Chlamydia*-%) from paired swab and liver samples. (XLSX)

S4 Table Results for MLST analysis and *omp*A genotyping for the 12 selected *C*. *psittaci* samples. (XLSX)
